# LncRNAs as a new regulator of chronic musculoskeletal disorder

**DOI:** 10.1111/cpr.13113

**Published:** 2021-09-08

**Authors:** Hesuyuan Huang, Dan Xing, Qingxi Zhang, Hui Li, Jianjing Lin, Zihao He, Jianhao Lin

**Affiliations:** ^1^ Arthritis Clinic & Research Center Peking University People's Hospital, Peking University Beijing China; ^2^ Arthritis Institute Peking University Beijing China

**Keywords:** arthropathy, chronic musculoskeletal disorder, long non‐coding RNA, rachiopathy, osteopathy

## Abstract

**Objectives:**

In recent years, long non‐coding RNAs (lncRNAs) have been found to play a role in the occurrence, progression and prognosis of chronic musculoskeletal disorders.

**Design and methods:**

Literature exploring on PubMed was conducted using the combination of keywords 'LncRNA' and each of the following: 'osteoarthritis', 'rheumatoid arthritis', 'osteoporosis', 'osteogenesis', 'osteoclastogenesis', 'gout arthritis', 'Kashin‐Beck disease', 'ankylosing spondylitis', 'cervical spondylotic myelopathy', 'intervertebral disc degeneration', 'human muscle disease' and 'muscle hypertrophy and atrophy'. For each disorder, we focused on the publications in the last five years (5/1/2016‐2021/5/1, except for Kashin‐Beck disease). Finally, we excluded publications that had been reported in reviews of various musculoskeletal disorders during the last three years. Here, we summarized the progress of research on the role of lncRNA in multiple pathological processes during musculoskeletal disorders.

**Results:**

LncRNAs play a crucial role in regulating downstream gene expression and maintaining function and homeostasis of cells, especially in chondrocytes, synovial cells, osteoblasts, osteoclasts and skeletal muscle cells.

**Conclusions:**

Understanding the mechanisms of lncRNAs in musculoskeletal disorders may provide promising strategies for clinical practice.

## Graphical abstract

With the advent of the aging society, the number of patients with chronic musculoskeletal disorders is increasing. Through studies, long non‐coding RNAs have the potential to become biomarkers and therapeutic targets of emerging musculoskeletal disorders.

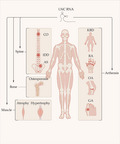

## 1 INTRODUCTION

Musculoskeletal disorders are a group of conditions that affect the motor system, including bones, muscles, tendons, ligaments and joints.^1^ People with multiple disorders are particularly vulnerable, especially in the context of an ageing population. Musculoskeletal disorders include a variety of conditions such as osteoarthritis (OA), rheumatoid arthritis (RA), osteopenia, osteoporosis, fractures, sarcopenia, etc.^2^

Non‐protein‐coding RNA makes up 98% of the whole human genome.^3^, ^4^ These functional RNAs can be divided into two groups according to the threshold of 200 nucleotides (NTS): small and long non‐coding RNAs (lncRNAs).^5^, ^6^ LncRNAs regulate the activities of both nearby and distant genes by multiple mechanisms. It could act as a scaffold for transcription factors and other molecules involved in transcription initiation.^7^ Moreover, it could serve as protein and microRNA decoys to interfere with cell division by regulating a series of key genes.^8^ For those mainly located in the cytoplasm, it could directly target mRNA and induce translation.^9^ Currently, an increased number of lncRNAs are found to be involved in the regulation of development and homeostasis of skeletal muscle system.^10^, ^11^ It is notable that lncRNAs take key roles in musculoskeletal disorders.

In this review, we summarized the functions and mechanisms of lncRNAs involved in the occurrence and progression of musculoskeletal disorders. Meanwhile, the potential of lncRNAs as promising targets for musculoskeletal disorders was also highlighted. An in‐depth study of the pathological process, molecular regulatory mechanisms, cytokines and therapeutic targets of musculoskeletal disorders would greatly benefit patients before they progress to the end stage of the disease. We hope that this review will provide insight into the potential of lncRNAs as biomarkers and therapeutic targets for musculoskeletal disorders.

## 2 LNCRNAS AND OSTEOARTHRITIS

### 2.1 Introduction of OA

OA, one of the most common musculoskeletal disorders, has been rising since the mid‐20th century. It usually begins with age‐related degeneration of the articular cartilage surface, and its main pathological feature is cartilage destruction. At the joint level, pathogenic factors include joint injury, joint dislocation, abnormal joint loading and other factors.^12^ It is well known that extracellular matrix (ECM) destruction,^13^, ^14^ inflammatory response and synovitis,^15^, ^16^ cell proliferation,^17^ cell death (including apoptosis and autophagy)^18^, ^19^ and angiogenesis^20^ are closely related to the pathological process of OA.

**FIGURE 1:**
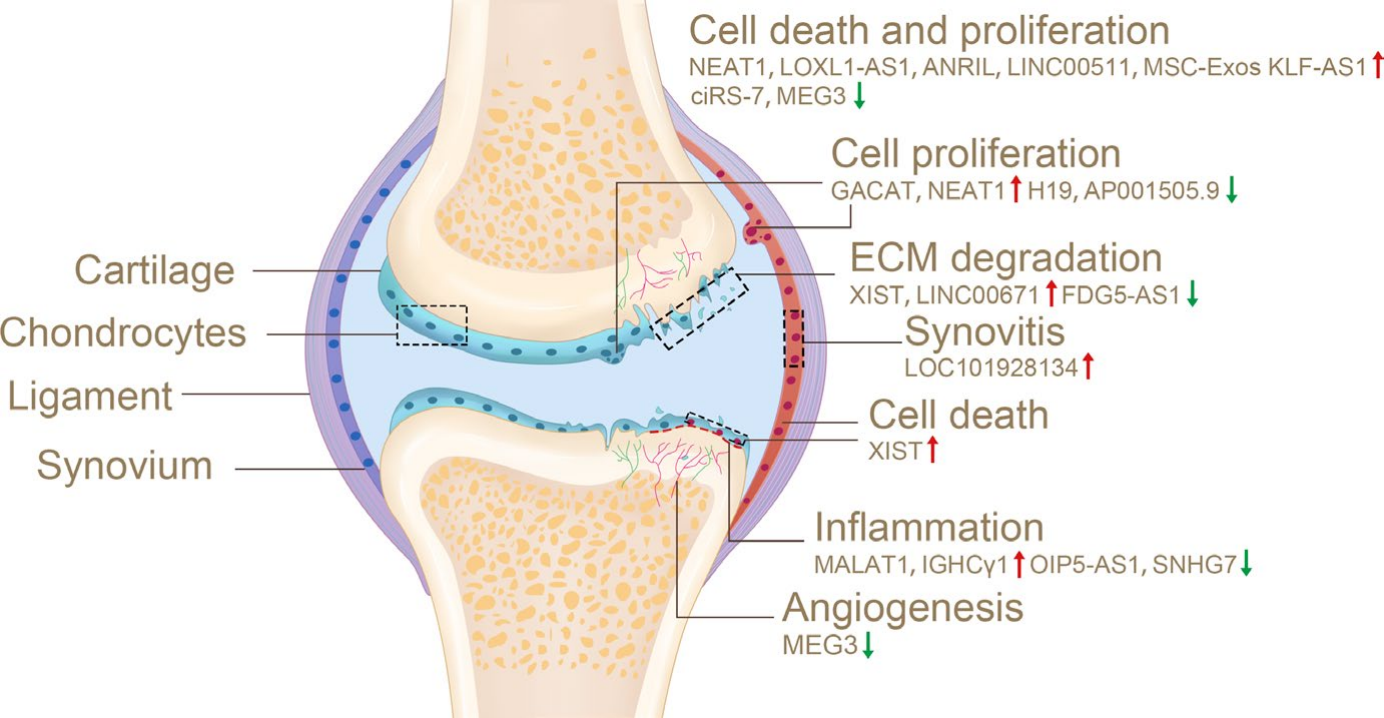
In OA, the function of lncRNA was proved by experiment. ECM degradation, inflammatory response, synovitis, angiogenesis, cell death and proliferation are known to promote the development of OA. Red arrows indicate upregulation, and green arrows represent downregulation

As early as in 2014, Xing et al reported the differentially expressed lncRNAs (73 up and 48 down) in OA cartilage compared with normal cartilage through microarray analysis.^21^ Mounting studies have shifted from merely concentrating on the fate of articular cartilage to evaluating how the intra‐articular microenvironment influences the occurrence and progression of OA. Detailed pathological process of OA is described in Figure 1. LncRNAs related to OA that have appeared in other literatures^22^, ^23^ will not be introduced in detail in this review. Together with the lncRNAs presented in this review, they are summarized in Table 1. This review mainly focuses on recent studies of lncRNAs.

**TABLE 1:** Summary of the roles of lncRNAs in OA

LncRNAs	Expression	Targets	Study models	Cellular process	Reference
XIST	Up	miR‐1277‐5p, MMP‐13/ADAMTS‐5	Human primary chondrocytes and rat cartilage	ECM degradation (+)	^27^
XIST	Up	TIMP‐3	Human primary chondrocytes	Collagen degradation (+)	^28^
LINC00671	Up	ONECUT2/Smurf2	Human primary chondrocytes and mice cartilage tissue	Cell proliferation (−), cell apoptosis and ECM degradation (+)	^29^
FGD5‐AS1	Down	miR‐302d‐3p/TGFBR2	Human cartilage tissue and human chondrocytes cell line(C20/A4)	Cell viability (+), apoptosis, and ECM degradation (−)	^30^
MALAT1	Up	PGE2/OPG	Human primary OA osteoblasts and serum	Inflammation (+)	^37^
OIP‐AS1	Down	miR‐29b‐3p/PGRN	Human cartilage tissue, CHON‐ 001(human chondrocyte cell line) and ATDC5(mouse chondrocyte cell line) and HEK293(human embryonic kidney cell line) cell	Proliferation and migration (+); apoptosis and inflammation (−)	^38^
SNHG7	Down	miR‐214‐5p; PPARγ1	Human and mice primary chondrocytes	NLRP3 inflammasome and apoptosis (−)	^39^
IGHCγ1	Up	miR‐6891‐3p/TLR4/NF‐κB	hPBMCs and THP‐1 monocytic cell line	Inflammatory response (+)	^40^
LOC101928134	Up	IFNA1; JAK/STAT signaling	Rat cartilage, synovium tissue and serum	Synoviocytes apoptosis (−) and chondrocytes apoptosis (+)	^41^
LOXL1‐AS1	Up	miR‐423‐5p/KDM5C; JUND1	Human primary chondrocytes	Proliferation and inflammation (+)	^52^
ciRS‐1	Down	miR‐7	Human peripheral blood and monocytes, C28/I2 chondrocytes cell line	Apoptosis and inflammation (−)	^53^
MEG3	Down	miR‐16/SMAD7	Rat primary chondrocytes, cartilage tissue and HEK 293T cell line	Proliferation (−) and apoptosis (+)	^54^
XIST	Up	miR‐376c‐5p/OPN	Human primary chondrocytes and THP‐1 cell line	Inflammatory microenvironment and apoptosis (+)	^55^
XIST	Up	miR‐149‐5p/DNMT3A	Human and rat cartilage tissue, human chondrocyte cell line (CHON‐001)	Cell viability (−), apoptosis and ECM degradation (+)	^57^
XIST	Up	miR‐142‐5p/SGTB	Human chondrocytes cell line (SW1353) and HEK 293T cell line	Proliferation (−) and apoptosis (+)	^58^
KLF‐AS1	Up	‐	Human MSC (exosomes) and rat primary chondrocytes and cartilage tissue	Proliferation (+) and apoptosis (−)	^59^
KLF‐AS1	Up	miR‐206/GIT1	Human MSC (exosomes) and rat primary chondrocytes	Proliferation (+) and apoptosis (−)	^60^
H19	Down	miR‐106b‐5p/TIMP2	Human cartilage tissue; rat primary chondrocytes and FLS	Chondrocyte proliferation and migration (+); matrix degradation (−)	^61^
GACAT3	Up	IL‐6/STAT3	Human synoviocytes	Proliferation (+) and apoptosis (−)	^62^
ANRIL	Up	miR‐122‐5p/DUSP4	Human cartilage tissue and human synoviocytes	Proliferation (+) and apoptosis (−)	^63^
LINC00511	Up	miR‐150‐5p/SP1	The mouse chondrocytic cell line (ATDC5)	Proliferation (−), apoptosis (+) and ECM synthesis of chondrocyte (+)	^64^
NEAT1	Up	miR‐543/PLA2G4A	Human primary chondrocytes	Proliferation (−) and apoptosis (+)	^65^
NEAT1	Up	miR‐181c/OPN	Human primary synoviocytes	Proliferation (−) and inflammatory (+)	^56^
MEG3	Down	VEGF	Human cartilage tissue	Angiogenesis (−)	^68^

Abbreviations: ciRS‐1, conserved inverted repeat sequences‐1; ECM, extracellular matrix; FLSs, fibroblast‐like synoviocytes; GACAT3, gastric cancer associated transcript 3; GIT1, G‐protein‐coupled receptor kinase interacting protein‐1; HRAS, Harvey rat sarcoma viral oncogene homolog; IL‐6, Interleukin‐6; JAK‐STAT, Janus kinase‐signal transducer and activator of transcription; KDM5C, lysine demethylase 5C (KDM5C); LOXL1‐AS1, lysyl oxidase like 1 antisense RNA 1; MAPK, mitogen‐activated protein kinase; MEG3, Maternally expressed gene 3; NEAT1, nuclear‐enriched abundant transcript 1; NF‐κB, nuclear factorκB; NLRP3, the nucleotide‐binding oligomerization domain‐like receptor family pyrin domain‐containing 3; OIP5‐AS1, OIP5 antisense RNA1; ONECUT2, one cut homeobox 2; OPN, osteopontin; PBMC, peripheral blood monouclear cell; PGRN, progranulin; SNHG7, Small nucleolar RNA hostgene 7; STAT3, signal transducer and activator of transcription 3; TIMP2, tissue inhibitor of metalloproteinase‐2; TIMP‐3, Tissue inhibitor of metalloproteinase‐3; TLR4, toll‐like receptor 4; XIST, long noncoding RNA X‐inactive specific transcript.

### 2.2 Role of lncRNAs in ECM degradation in OA

Articular cartilage is a type of connective tissue made up of chondrocytes. But, interestingly, chondrocytes make up only 1% of normal cartilage volume. The auto‐synthetic ECM blocks the chondrocytes. Nonetheless, they provide mechanical support for the cartilage and lubrication of the joint. It is also responsible for the composition and integrity of the matrix.^24^ In OA chondrocytes, matrix metalloproteinases (MMPs) (including MMP‐1 and MMP‐13), metalloproteinase with a thrombospondin type 1 motif (ADAMTS) (including ADAMTS 1,4,5) and various types of disintegrin have been found to significantly increase the expression of matrix degrading proteins.^25^ In addition, fibroblast‐like synoviocytes (FLSs) has been reported to overexpress several enzymes (such as MMP‐13) that degrade ECM.^26^

Due to the unique composition of cartilage, ECM degradation is the most popular mechanism of OA associated with lncRNA. Recent studies have shown that lncRNA XIST (long non‐coding RNA X‐inactive‐specific transcript) can be regarded as a star lncRNA. XIST was revealed to be upregulated in OA specimens and articular chondrocytes derived from OA tissue and IL‐1β‐treated articular chondrocytes (ACs). Downregulation of XIST suppresses the degradation of the ECM by binding a competing endogenous RNA (ceRNA) of miR‐1277‐5p.^27^ LncRNA XIST is mainly localized in the nucleus and could bind to the promoter of tissue inhibitor of metalloproteinase‐3 (TIMP‐3). Silencing of XIST reduced the methylation level of TIMP‐3 promoter and increased TIMP‐3 expression, thereby inhibited collagen degradation in OA chondrocytes. It can rapidly recruit and maintain DNA methyltransferase DNMT1, induce the number of new methyltransferases DNMT3A and DNMT3B and down‐regulate the expression of TIMP‐3. XIST could block the further collection and binding of the promoter region of TIMP‐3 and improve the methylation rate of its CpG island.^28^

LINC00671 induces ubiquitination of GSK‐3β, an important regulator of MMP‐mediated joint destruction, and enhances β‐catenin expression through Smurf2. Mechanically, its inhibition may enhance endochondral ossification and mitochondrial oxidative stress, increase cell death and β‐catenin expression and ultimately lead to ECM remodelling.^29^ Defects of the TGF‐β signalling pathway may make cartilage more susceptible to damage. The typical TGF‐β signalling pathway is activated by three TGF‐β subtypes, including type II serine/threonine kinase receptors (TGFBR2). FGD5‐AS1 protected chondrocytes from damage caused by inflammation and reduced ECM degradation through miR‐302D‐3p/TGFbR2 axis.^30^

### 2.3 Role of lncRNAs in Inflammation and synovitis in OA

Inflammatory manifestations of OA are usually confined to adjacent areas of pathologically damaged cartilage and bone.^31^, ^32^ Chondrocytes treated with IL‐1β are commonly used to simulate OA chondrocytes, demonstrated suppressed proliferation, increased apoptotic rates and differential expression of type II collagen alpha 1(COL2A1) and MMP‐13.^33^, ^34^ Synovial inflammation is caused by a large number of soluble inflammatory mediators, including IL‐1β and tumour necrosis factor‐α (TNF‐α), two major cytokines involved in the pathogenesis of OA.^35^ Supporting these conclusions, multiple pro‐inflammatory cytokines have been detected at higher levels in serum and synovial fluid in OA patients than in those of healthy individuals. Importantly, the effects of related cytokines on cartilage and bone tissue demonstrated in vitro were similar to the structural changes observed in OA joints in vivo.^36^

In OA subchondral bone, MALAT1 has been proved to be highly expressed. Human OA osteoblasts induced expression of MALAT1 and regulate PGE2 production under inflammatory stimulation. PGE2 secretion is significant in OA osteoblasts that are MALAT1‐depleted and IL‐1β‐induced inflammation. PGE2 sensitises nociceptors through class E prostaglandin receptors (EP2 and EP4). This may cooperate with IL‐1β to induce the expression of IL‐6 and iNOS. In addition, MALAT1 may be closely related to inflammatory pain in OA.^37^ Zhi et al suggested that that the expression of miR‐29b‐3p was decreased and the expression of PGRN was significantly increased in OA model, because miR‐29b‐3p might bind to the 3 '‐UTR of PGRN. In addition, after the elimination of lncRNA OIP5‐AS1, the expression of PGRN in OA model was decreased.^38^ MiR‐214‐5p is overexpressed in patients with OA, and it enhances IL‐1β‐induced chondrocyte inflammation. However, SNHG7 attenuated the release of NLRP3 inflammasomes and apoptosis of chondrocytes. The possible mechanism is that SNHG7 sponges miR‐214‐5p, which targets the 3′‐untranslated region (UTR) of PPAR gamma‐coactivator‐1beta (PGC‐1β).^39^ In OA, lncRNA IGHCγ1 was upregulated and was mainly localized in macrophage cytoplasm. LncRNA IGHCγ1 promotes the expression of TLR4 (Toll‐like receptor 4) by acting as a ceRNA of miR‐6891‐3p through NF‐κB signalling in macrophages. Meanwhile, by targeting TLR4, miR‐6891‐3p inhibited the inflammatory response of macrophages, and the proliferation and migration of macrophages.^40^ According to the database, LOC101928134 is located in the region of chromosome 15q13.3. One of the interferons (IFN) was also identified at 15q13.3. IFN are a class of glycoproteins commonly known as cytokines produced by immune cells. Downregulation of LOC101928134 can reduce knee synovitis, inflammation and knee cartilage injury in OA rats by regulating the expression of IFNA1 and restraining the JAK/STAT (Janus kinase/signal transducers and activators of transcription) signalling pathway.^41^

### 2.4 Role of lncRNAs in cell death and proliferation in OA

Unregulated apoptosis, autophagy and cell necrosis constitute the injuries of chondrocyte.^42^, ^43^ The ratio, HIF‐1α/HIF‐2α, is the main regulator of chondrocyte survival and death, and it alters the balance of apoptosis or autophagy. Moreover, death of chondrocytes showed a periodic pattern under the influence of Fas, SNPs, pro‐inflammatory cytokines and mechanical constraints, involving mitochondrial dysfunction, ROS production, p38 activation and Bcl‐2/Bax ratio.^44^ Several autophagy and mitophagy‐related proteins (such as LC3B, SQSTM1 and PINK1) have been found to be highly expressed in human OA cartilage and monosodium iodoacetate (MIA)‐induced rodent models of OA.^45^ Among the mechanisms of chondrocyte injury, apoptosis and autophagy are the main research focuses to study the pathogenesis of OA and identify potential therapeutic targets, because these processes are mainly regulated by the cell itself.

Although chondrocytes proliferation is associated with natural regeneration, it may also lead to pathological processes.^46^ Chondrocytes proliferate actively, causing some of them to grow and others to undergo hypertrophic changes to become hypertrophic chondrocytes.^47^ On the molecular level, chondrocyte hypertrophy differentiation may also be characterized via high expression of collagen type X, MMP13 and Runt‐associated transcription factor 2 (Runx2). Hyaline cartilage markers are decreased in the hypertrophic cells. It contains collagen type II, aggrecan and SOX9.^48^ Some evidences indicate that OA‐derived FLSs (OA‐FLSs) play an important role in the proliferation, migration and apoptosis of chondrocytes.^49^, ^50^ Nuclear expression of p16 is highly expressed in synovial tissues of OA, suggesting senescence of synovial fibroblasts. Ageing synovial fibroblasts induced by H_2_O_2_ or TNF‐α express CDKN1A and CDGN2A as well as other pro‐inflammatory SASP‐related factors.^51^

Certain lncRNAs play a vital role in both cell death and proliferation. Generally, lncRNA regulates cell behaviours by targeting cyclins related to the cell cycle, cyclin‐dependent kinase (CDK) and/or its inhibitors.^8^ Downregulation of LOXL1‐AS1 significantly inhibited the proliferation of OA chondrocytes, but promoted apoptosis.^52^ In the process of OA, ciRS‐7/miR‐7 axis may play a regulatory role in mediating chondrocyte apoptosis, proliferation and inflammatory response.^53^ In the rat chondrocytes induced by IL‐1β, MEG3 gene knockdown can promote proliferation and inhibit apoptosis, while miR‐16 gene knockdown can inhibit proliferation and promote apoptosis.^54^

A recent study revealed that macrophage synovial cells, once activated by the inflammatory microenvironment in OA, secrete pro‐inflammatory cytokines, degrading enzymes and adhesion molecules that accelerate chondrocyte apoptosis and cartilage degradation. XIST acts as a ceRNA against OPN (osteopontin), making it bind to miR‐376C‐5p, thus counteracting the OPN inhibition mediated by miR‐376C‐5p. OPN has been reported to regulate expression of various factors associating with the pathogenesis of OA, including MMP13, hypoxia‐inducible factor‐2α (HIF‐2α), ADAMTS4, TIMPs, IL‐6 and 8, and even caveolin‐1. Co‐culture with M1 macrophages overexpressing OPN can significantly inhibit chondrocyte migration and significantly increase chondrocyte apoptosis. OPN overexpression enhances the cytotoxicity of M1 macrophages to chondrocytes by regulating chondrocyte apoptosis and ECM degradation.^55^, ^56^ Another report showed that overexpression of DNMT3A inhibited apoptosis and ECM degradation, but reduced miR‐149‐5p‐induced cell viability promotion. This explains why XIST knockout can inhibit the development of OA through the miR‐149‐5p/DNMT3A axis.^57^ The inhibition of SGTB expression by miR‐142‐5p could be relieved by lncRNA XIST. Therefore, the inhibition of miR‐142‐5p or the enhancement of SGTB can reverse the effects of XIST deletion on the growth and apoptosis of chondrocytes.^58^

Exosomes are described as a kind of extracellular vesicles secreted by MSC in a resting state or under certain types of stress (such as hypoxia, radiation or oxidative damage), which can act as a messenger between MSC and differentiated cells, thereby inducing physiological changes. It has previously been reported that exosomes secreted from human MSCs promote cartilage regeneration. Exosomes lncRNA KLF3‐AS1 promote cartilage repair model of OA rats.^59^ In another study by the same group, it is involved in apoptosis inhibition and proliferation induction of chondrocyte via the miR‐206/GIT1(G‐protein‐coupled receptor kinase interacting protein 1) axis.^60^ Similarly, FLS‐derived exosomes also play a role in the pathological process of OA. The enhancement of cell proliferation and migration during exosome‐mediated cartilage repair is related to the regulation of miR‐106b‐5p/TIMP2 axis mediated by exosomal lncRNA H19.^61^

A large number of osteoarthritis synoviocytes (OAS) could secrete cytokines to destroy the structure of bone and cartilage. GACAT3 affects OAS proliferation through the interleukin 6/signal transduction and transcriptional activator −3 (IL‐6/STAT3) signalling pathway.^62^ In addition, the expression of ANRIL is decreased and cell proliferation is reduced in OAS. The cell cycle is suspended in G0/G1 phase and cell apoptosis is improved in OAS. And the proliferation and apoptosis of OAS were regulated by ANRIL through the miR‐122‐5p/DUSP4 axis.^63^ Another study aimed to uncover knockdown of LINC00511 that facilitates proliferation and represses the apoptosis and ECM synthesis of chondrocytes. Mechanically, LINC00511 functions as a sponge for miR‐150‐5p and interacts with the 3'‐UTR region of transcription factor (SP1). In turn, transcription factor SP1 binds with the promoter region of LINC00511 and thus upregulates LINC00511 expression.^64^ Overexpression of NEAT1 inhibited p‐AKT1 and Bcl‐2 expression and upregulated ILs (6 and 8) and MMPs (3, 9 and 13). However, this effect of overexpression of NEAT1 could be reversed by miR‐543 simulants. NEAT1 can inhibit cell proliferation and promote apoptosis via miR‐543/PLA2G4A axis.^65^ NEAT1 and OPN competed for the binding of miR‐181c. Subsequently, the inhibitory effect of miR‐181c on synovial cell proliferation and related factors inhibited by NEAT1 knockdown could be partially reversed.^56^

In a novel study, researchers characterized the lncRNA expression profiles in human hyaline chondrocyte dedifferentiation, thereby identifying new potential mechanisms of chondrocyte dedifferentiation. It was found that AP001505.9 overexpression inhibited the dedifferentiation of chondrocytes. This discovery paves the way for further investigation into the mechanisms of dedifferentiation and OA treatment.^66^

### 2.5 Role of lncRNAs in angiogenesis in OA

The first step in ossification is vascular invasion, usually in non‐vascular cartilage. The vascular system provides channels for different types of cells to participate in the recruitment of cartilage absorption and bone deposition.^67^ VEGF is involved in vascular invasion of growth plate cartilage, hypertrophic cartilage remodelling and ossification of growth plate cartilage.^20^ In the process of the development of OA, the level of MEG3 is negatively correlated with the level of VEGF, suggesting that MEG3 may regulate angiogenesis.^68^ Currently, the importance of angiogenesis in the aetiology of OA has been demonstrated. It has been revealed that inhibition of angiogenesis may be a potential therapeutic target for OA by reducing OA‐related pain and inflammation.^69^, ^70^

## 3 LNCRNAS AND RHEUMATOID ARTHRITIS (RA)

RA is a chronic systemic autoimmune disease of unknown aetiology. The typical clinical features of RA are symmetric peripheral arthritis and progressive erosion of the affected joints. If left untreated, the disease presents as persistent synovitis and erosion of articular cartilage and surrounding bone.^71^ It is worth noting that RA‐FLSs are critical to synovial aggression and joint destruction. And it may play a vital role in the occurrence and development of the disease.^72^ Through studies of human specimens, lncRNAs might be involved in the molecular pathophysiology of RA.^73^ In another RA study, analysis of exosomal lncRNAs identified several differentially expressed lncRNAs, including MALAT1, HOTAIR, MEG9, SNHG1, SNHG4, HOTAIR, TUG1 and NEAT1.^74^ Some lncRNAs have been identified by recognizing inflammatory pathways in RA, such as p38 MAPK, TLR and NF‐κB signalling pathways.^75^

H19 activates JNK/p38 MAPK and NF‐κB pathways by promoting TAK1 phosphorylation. And H19 knockdown obviously lowered the levels of IL‐8, IL‐1β and IL‐6, which was consistent with the above outcomes.^76^ The anti‐inflammatory ability of HOTTIP silencing is via the demethylation of the SFRP1 promoter in RA synovial fibroblasts (RASFs). By changing HOTTIP/Dnmt3b/SFRP1 expression in RASFs, the regulatory mechanism was explored. It was noted that HOTTIP SFRP1 can be induced by promoter methylation, Dnmt3b recruitment and activation of the Wnt‐signalling pathway.^77^ The role of MEG3 in RA may be related to the regulation of miR‐141 and Akt/mTOR signalling pathways by increasing the proliferation rate.^78^

Li et al uncovered that MALAT1 binds to the CTNNB1 promoter and modulates DNA methylation to inhibit β‐catenin and Wnt‐signalling pathway. MALAT1 from exosomes has also been shown to regulate RASF proliferation and inflammatory response by increasing the secretion of TNFα, IL‐6 and IL‐10.^79^ Researchers have studied the mechanism of NEAT1 in RA development and found it acted by modulating the miR‐23a/MDM2 (murine double minute 2)/SIRT6 axis through PBMC‐exos (peripheral blood monouclear cell‐derived exosomes). During the pathogenesis of RA, SIRT6 is degraded by ubiquitination of MDM2. LncRNA NEAT1 promotes FLS proliferation and inflammatory response by regulating the MDM2/SIRT6 axis through PBMC‐derived exos. Furthermore, in vivo experiments have shown that downregulation of lncRNA NEAT1 or upregulation of miR‐23a via PBMC‐derived outer membrane transportation can alleviate the deterioration of RA in mice.^80^

In addition to the pro‐proliferative and anti‐apoptotic roles of exosomal NEAT1, the upregulation of NEAT1 promotes migration, invasion and inflammatory cytokine secretion in RA‐FLSs.^81^ By reducing FLS synovitis in RA, silencing of NEAT1 can promote miR‐129 and miR‐204 to repress the ERK/MAPK signalling pathway. At the same time, it can also target miR‐204‐5p through the NF‐κB pathway to attenuate TNFα‐induced FLS proliferation and production of inflammatory cytokines, while promoting apoptosis.^82^, ^83^ In RA‐FLS, SNHG1 helps maintain cell proliferation, migration and invasion functions. Furthermore, the modulation mechanism depends on the interaction between the SNHG1 and polypyridine binding protein 1 (PTBP1).^84^ ZFAS1 is involved in the progression of RA by competitively binding miR‐296‐5p and regulating the expression of MMP‐15.^85^ Linc‐PINT inhibits TNF‐α‐induced cell proliferation and invasion. This may be caused by downregulation of miR‐155‐5p, modifying the expression of SOCS1, IL‐1β and MMPs, as well as the inactivation of ERK signalling pathway.^86^

Based on existing literatures, several non‐coding RNAs have been shown to be dysregulated in different samples from RA patients, but the dysregulated RNAs in serum are the most suitable biomarkers for use. The lncRNAs discussed in this section are summarized in Table 2, and the lncRNAs that appear in these reviews^87^, ^88^, ^89^ are excluded, such as PISCAR,^90^ LERFS^72^ and NTT.^91^

**TABLE 2:** Summary of the roles of lncRNAs in RA

LncRNAs	Expression	Targets	Study models	Cellular process	References
H19	Up	TAK1; NF‐κB and JNK/p38	Human synovial cell line (MH7A)	Release of inflammatory cytokines (+)	^76^
HOTTIP	Up	Dnmt3b/SFRP1	Human primary RASFs and OASFs; Rat synovial tissue	Proliferation, invasion, and migration (+); apoptosis (−); inflammation (+)	^77^
MEG3	Down	miR‐141; AKT/mTOR	Rat primary chondrocytes and cartilage tissue	Proliferation (+) and inflammation (−)	^78^
MALAT1	Down	CTNNB1	Human primary FLS	Proliferation and inflammation (−)	^79^
NEAT1	Up	miR‐23a/MDM2/SIRT6	Human PBMCs (exosomes), mice primary FLSs and synovial tissue	Proliferation and inflammation (−)	^80^
NEAT1	Up	miR‐410‐3p/YY1	Human synovial tissue, hFLS and hFLS‐RA cell lines	Migration, invasion, and inflammatory cytokines secretion (+)	^81^
NEAT1	Up	miR‐129/miR‐204; MAPK/ERK	Human and rat synovial tissue, human peripheral blood and rat primary FLSs	Proliferation of FLSs, and synovitis (+)	^82^
NEAT1	Up	miR‐204‐5p/NF‐κB	RA‐FLS cell line and human synovial tissue	Proliferation and inflammatory cytokine production (+)	^83^
SNHG1	Up	PTBP1	Human primary FLSs	Proliferation, migration and invasion (+)	^84^
ZFAS1	Up	miR‐296‐5p; MMP‐15	Human synoviocyte MH7A cell line; Mice synovial tissue and blood	Proliferation (−) and apoptosis (+)	^85^
PINT	Down	miR‐155‐5p/SOCS1; ERK	Human primary FLSs	Proliferation and invasion (−)	^86^

Abbreviations: TAK1, transforming growth factor beta‐activated kinase 1; Dnmt3b, DNA methyltransferase 3b;SFRP1, secreted frizzled‐related protein 1; RA/OASF, RA/OA synovial fibroblasts; mTOR, mechanistic target of rapamycin; CTNNB1, β‐catenin; MDM2, mouse double minute 2; SIRT6, Sirtuin 6; YY1, the transcription factor Yin Yang 1; PTBP1, polypyrimidine tract binding protein 1; MMP15, matrix metalloproteinase 15; PINT, premature Infants in Need of Transfusion; SOCS1, suppressor of cytokine signalling 1; ERK, extracellular regulated protein kinases.

## 4 LNCRNAS AND OSTEOPOROSIS

Osteoporosis results from the disruption of the balance between osteoblast‐mediated bone formation and osteoclast‐mediated bone resorption. Osteoporosis is a chronic systemic bone disorder characterized by loss of bone mass, microstructural destruction and increased fragility. One investigation found that more than one third of women over 50 years of age have osteoporosis, while only one fifth of men have osteoporosis,^92^ indicating that women are at higher risk of osteoporosis than men. Before the age of 30, the process of bone loss begins. And it continues until death as a by‐product of ageing. Release of inflammatory factors such as TNF and IL‐6 by senescence cells, as well as changes in the composition of bone marrow cells (osteoclast precursors, monocytes and granulocytosis), contributes to osteoporosis in the elderly.^93^

### 4.1 The role of LncRNAs in osteogenesis

Through competing endogenous RNA networks, we have identified functional lncRNAs in osteoblastic differentiation.^94^ Long non‐coding RNAs may serve as regulators of bone marrow stem cells (BMSCs) in osteoporosis.^95^ The differentiation from MSCs to osteoblasts is a precise process regulated by multiple signalling pathways.^96^, ^97^, ^98^ Many studies have shown that the expression profile of lncRNA changes dynamically during osteogenic differentiation.

BMSCs are the main source of osteoblasts, which are widely used in bone remodelling and bone regeneration. Osteogenic differentiation of BMSCs is synergically promoted by H19 and FoxC2 through the Wnt‐β‐catenin pathway.^99^ Researchers confirmed that supplementing aged BMSCs with lnc‐PMIF knockdown mediated by small interfering RNA (siRNA) can promote bone formation in aged mice. Mechanistically, LNC‐PMIF can bind human antigen R (HuR) to block the interaction of HuR‐β‐actin mRNA, thereby inhibiting the expression of β‐actin and inhibiting the migration of OPCs (osteoprogenitor cells) in the elderly.^100^ LncRNA NKILA plays an important positive regulatory role in the process of osteogenesis of MSCs, and its knockdown significantly inhibited the osteogenesis of menstrual blood‐derived mesenchymal stem cells (MENSCs) and umbilical cord mesenchymal stem cells (UCMSCs).^101^ HOTAIR is an essential regulator of BMP9‐induced osteogenesis of MSCs in the murine family, acting by targeting cell cycle and proliferation.^102^ Through FBXO25/H2BK120ub H3K4me3/OSX axis, ODIR1 plays a negative regulatory role in the osteogenic differentiation of hUC‐MSCs.^103^ Intravenous administration of siHOXC‐AS3 has been shown to be effective in preventing bone loss through its anticatabolic activity and bone formation in a mouse model. This result suggests that lncHOXC‐AS3 promotes bone formation of BMSCs by enhancing HOXC10 expression.^104^ Another study presented a new mutual effect between STAT3 and LINC02349. Furthermore, LINC02349 acts as a spongy RNA for miR‐33b‐5p and miR‐25‐3p, regulating Smad5 and Wnt10b. Thus, the osteogenic differentiation of hUC‐MSCs can be regulated.^105^ LncRNA ENST00000563492 promotes the osteogenic differentiation of BMSCs by upregulating the expression of CDH11. During this process, the expression of VEGF improves the coupling process of osteogenesis and angiogenesis.^106^ Studies have revealed that MIR22HG expression is significantly reduced in BMSCs of osteoporotic mice and upregulated in hBMSCs during osteogenic differentiation.^107^ In addition, a considerable quantity of literatures have described MSCs have the abilities not only in osteogenic differentiation, but also in adipogenic, myogenic and chondrogenic differentiation.

The basic pathogenesis of postmenopausal osteoporosis (PMOP) is excessive bone resorption and insufficient bone formation due to oestrogen deficiency.^108^ Here, we summarized the following related studies on PMOP and lncRNAs. In the model of PMOP, BMSCs show a loss of viability and pluripotency. Downregulation of LNC_000052 promoted proliferation, migration and osteogenesis of BMSCs and inhibited apoptosis via miR‐96‐5p/PIK3R1 axis.^109^ Studies have shown that iron accumulation (IA) is closely related to PMOP. Consistent with the performance of inhibiting XIST, in the IA model, miR‐758‐3p mimic reduced caspase 3 activation, osteoblast apoptosis and osteoporosis symptoms.^110^ Mediated by miR‐532‐3p/SIRT1 signalling, LncRNA H19 in BMSCs regulates oestrogen‐regulated osteogenic differentiation.^111^ Previous studies have shown that under the condition of ER stress, the significantly reduced expression of TIMP1 is correlated with the increased apoptosis of osteoblasts.^112^ Another study has shown that endoplasmic reticulum stress and miR‐138 expression can both activate the osteoblastic apoptosis pathway. Oestrogen deficiency can induce apoptosis of osteoblasts in postmenopausal women and lead to osteoporosis by regulating HOTAIR/miR‐138/TIMP1 signalling axis.^113^ Understanding the epigenetic modifications of these lncRNAs and their regulatory roles will bring us closer to the potential gene modification therapy of PMOP for disease.

A study identified LncRNAs play an important role in NELL‐1‐induced osteogenesis of human adipose‐derived stem cells (hASCs) through crosstalk of Hedgehog and Wnt pathways. It was found that 323 lncRNAs were expressed differentially during osteogenesis and during NELL‐1‐induced osteogenesis.^114^

### 4.2 The role of LncRNAs in osteoclastogenesis

Osteoclasts are multinucleated cells that originate from monocyte/macrophage precursor cells and are responsible for bone resorption.^115^ The regulatory roles of lncRNAs in osteoclasts have been less studied than those in osteoblasts. The first study that systematically analysed the expression profile of lncRNAs at different stages of osteoclastogenesis was conducted by Dou et al in 2016.^116^

Data from previous studies confirmed a new signalling cascade in disuse osteoporosis (DOP): mechanical unloading causes the upregulation of DNMT1 and methylation of the H19 promoter, and ultimately leads to downregulation of H19 and inhibition of ERK signalling.^117^ Overexpression of PGC1β‐OT1 (peroxisome proliferator‐activated receptor γ coactivator‐1β‐OT1) in progenitor cells stimulates osteogenic differentiation. However, silencing of PGC1β‐OT1 inhibits mice osteogenic differentiation. PGC1β‐OT1 enhances the effect of KDM6B by antagonizing miR‐148a‐3p and reversely regulates osteogenic differentiation.^118^ Researchers have exploited the exosomal location of lncRNA‐MALAT1 in endothelial progenitor cells (EPCs) to promote the osteoclastic differentiation of bone marrow‐derived macrophages (BMMs). Mice treated with BMMs plus EPC‐derived exosomes showed increased neovascularization at the fracture site and enhanced fracture healing compared to mice treated with BMMs alone.^119^ Similar to osteoblast, osteoclast activity was enhanced by BMSCS‐derived exosome. MALAT1 from BMSCs‐derived exosomes may be used as a miR‐34c sponge to upregulate the expression of SATB2, contributing to the enhancement of osteogenic activity and the alleviation of osteoporosis symptoms in mouse models.^120^ Studies in vivo and in vitro have shown that the expression of NEAT1 is closely related to the formation of osteoclasts. Mechanically, NEAT1 competitively binds to miR‐7 and blocks its regulatory function of protein tyrosine kinase 2 (PTK2). Intergenic SNP rs12789028 acts as an allele‐specific long‐range enhancer of NEAT1 through chromatin interaction.^121^ In order to prevent the degradation of its target gene Smurf2, lncRNA CCAT1 could competitively bind to miR‐34a‐5p. Inhibitory CCAT1 improved the pathological state of osteoporotic rats in vivo and restricted the osteocyte apoptosis of bone tissue in vivo.^122^

Table 3 summarizes the lncRNAs introduced in this section. And it is excluded repeated parts of previous reports,^123^, ^124^, ^125^ such as DANCR,^126^ ORLNC1^127^ and XIST.^128^ Researchers have systematically summarized these lncRNAs.

**TABLE 3:** Summary of the roles of lncRNAs in Osteoporosis

LncRNAs	Expression	Targets	Study models	Cellular process	Reference
H19	Down	Foxc2; Wnt‐β‐catenin	Human serum and mice BMSCs	BMSCs osteogenic differentiation (+)	^99^
PMIF	Up	HuR; β‐actin	Mice bone, BMSCs and OPCs, MC3T3‐E1 clone 14 cell line, hFOB1.19 cell line	Aged OPCs migrating to bone formation surface (+), bone formation (−)	^100^
NKILA	Up	RXFP1/PI3K‐AKT and NF‐κB/ RUNX2	Human MenSCs and UCMSCs	Osteogenesis of MSCs (+)	^101^
mHOTAIR	–	–	Nude mice bone, iMAD‐MSCs, HEK‐293 and 293pTP cell lines	MSC osteogenesis (+)	^102^
ODIR1	Down	FBXO25/H2BK120ub/H3K4me3/OSX	hUC‐MSCs line (QC1205); HEK293 and 293 T cell lines; nude mice skin	Osteogenic differentiation (−)	^103^
HOXC‐AS3	Up	HOXC10	Human myeloma cell line(U266); Human MM‐MSCs; NSG mice	Osteogenesis of MM‐MSCs (−)	^104^
Linc02349	Up	miR‐25‐3p/miR‐33b‐5p/SMAD5/Wnt10b; Dlx5/OSX; STAT3	hUC‐MSCs line (QC1205); HEK293 and 293 T cell lines	Osteogenic differentiation (+)	^105^
ENST00000563492	Down	miR‐205‐5p/CDH11/VEGF	Human bone tissue, hBMSCs and HUVEC	Osteogenic differentiation of BMSCs (+)	^106^
MIR22HG	Down	PTEN/AKT	H and mBMSCs, hASCs, RAW264.7 cell line and OVX mice bone	Osteogenic differentiation of human BMSCs (+) and osteoclastogenesis of RAW264.7 cells (+)	^107^
LNC_000052	Down	miR‐96‐5p/PIK3R1	Rat BMSCs and OVX rat bone	BMSC proliferation, migration, osteogenesis (+) and apoptosis (−)	^109^
XIST	Up	miR‐758‐3p/caspase 3	IA mice plasma and bone; human osteoblasts (hFOB1.19 cell line)	Osteoblast apoptosis (+)	^110^
H19	Down	miR‐532‐3p/SIRT1	PMOP human bone and serum; OVX rat femur; hBMSCs	Estrogen‐regulated osteogenic differentiation in BMSCs (−)	^111^
HOTAIR	Down	miR‐138/TIMP1	PMOP human bone tissue; HFOB and MG63 cell lines	Estrogen‐regulated apoptosis of osteoblasts (−)	^113^
H19	Down	DNMT1; MAPK/ERK	Rat bone tissue; rat osteoblast cell line (UMR‐106); HEK 293T cell line	The development of DOP in HLU rats (−)	^117^
PGC1β‐OT1	–	miR‐148a‐3p/KDM6B	ST2, C3H10T1/2, and MC3T3‐E1 cells; mice MSCs and bone	Adipogenic(−) and osteogenic(+) differentiation	^118^
MALAT1	Up	miR‐124/ITGB1	Mice EPCs (exosomes), primary BMMs and mice bone tissue	Bone repair by enhancing recruitment and differentiation of osteoclast precursors (+)	^119^
MALAT1	Up	miR‐34c/SATB2	hBMSCs (exosomes); human osteoblasts (hFOB1.19) and OVX mice bone tissue	Osteoblast activity (+)	^120^
NEAT1	–	miR‐7/PTK2	293T cell lines, BMMs and mice bone and serum	Osteoclastogenesis (+) and bone mass (−)	^121^
CCAT1	Up	miR‐34a‐5p; SMURF2	OVX rats bone tissue and serum and rat primary osteoblasts	Osteoblasts proliferation and differentiation (−)	^122^

Abbreviations: BMMs, bone marrow derived macrophages; BMSC, marrow mesenchymal stem cell; CCAT1, colon cancer‐associated transcript 1; CDH11, cadherin‐11; DLX5, Distal‐less homeobox 5; DNMT1, DNA methyltransferase 1; DOP, disuse osteoporosis; EPCs, epithelial cells; ERK, extracellular signal‐regulated kinase; FBXO25, F‐box protein 25; Fox2, Forkhead box protein C2; h and mMSCs, human and mouse MSCs; H2BK120Ub, mono‐ubiquitination of histone H2B on lysine 120; H3K4me, Methylation of histone H3 lysine 4; hASCs, human adipose‐derived stem cells; HLU, hindlimb unloading; hUC‐MSCs, human umbilical cord‐derived mesenchymal stem cells; HUVECs, Human umbilical vein endothelial cells; IMADs, immortalized mouse adipose‐derived cells; ITGB1, integrinbeta1; KDM6B, Lysine‐specific demethylase 6B; MALAT1, metastasis‐associated lung adenocarcinoma transcript 1; MenSCs, menstrual blood‐derived mesenchymal stem cells; mHOTAIR, murine HOX transcript antisense RNA; MM‐MSCs, Bone marrow mesenchymal stem cells of multiple myeloma patients; NEAT1, nuclear‐enriched abundant transcript 1; NSG mice, NOD‐Prkdcscid Il2rgtm1/Bcgen mice; OSX, osterix; OVX, ovariectomized; PGC1β‐OT1, peroxisome proliferator‐activated receptor γ coactivator‐1β‐OT1; PI3K, phosphatidylinositol 3‐kinase; PI3KR1, phosphoinositide‐3‐kinase regulatory subunit alpha; PTEN, phosphatase and tensin homolog deleted on chromosome 10; PTK2, protein tyrosine kinase 2; RUNX2, Runt‐associated transcription factor 2; RXFP1, relaxin family peptide receptor 1; SATB2, Special AT‐rich sequence binding protein 2; SMURF2, smad ubiquitination regulatory factor 2; UCMSCs, umbilical cord mesenchymal stem cells; VEGF, vascular endothelial‐derived growth factor.

## 5 LNCRNAS AND GOUT ARTHRITIS (GA)

GA, the most common form of inflammatory arthritis, is caused by deposits of monosodium urate monohydrate (MSU) crystals in and around the joints. Elevated serum uric acid levels are considered to be an important risk factor for GA.^129^ It is well established that MSU causes inflammation in the pathological process of gout,^130^ and we are accustomed to refer to it as the key regulator of bone erosion in gout. Previous studies have identified several risk genes (SLC2A9, ABCG2 and URAT1) that are associated with elevated serum uric acid concentrations, thereby increasing the risk of developing GA.^131^, ^132^, ^133^ Significant changes in lncRNA H19 and ANRIL levels have been reported in patients with hyperuricemia and chronic kidney disease at high concentrations of uric acid. Liu et al found that H19 played a promoting role in renal tubular epithelial cell damage induced by CaOX nephrocalcinosis and kidney CaOX crystal deposition induced by glyoxylic acid. As a ceRNA, H19 acts through sponge‐mediated targeting of miR‐216b and through the HMGB1/TLR4/NF‐κB pathway. It was found that ANRIL promoted NLRP3 inflammasome activation through the miR‐122‐5p/BRCC3 axis in uric acid nephropathy (UAN).^134^, ^135^ In gouty arthritis monocytes, knockdown of HOTAIR significantly increased the expression of miR‐20b in the THP‐1 cell line stimulated by MSU and decreased the secretion of IL‐1β, NLRP3 and TNF‐α.^136^ LncRNA‐Jak3‐knockdown eliminated the formation of mature osteoclasts induced by MSU. Level of Jak3 in the monocytes of patients with gout is elevated. The activation of Nfatc1 mediated by LncRNA‐Jak3 upregulates the expression of cathepsin K (Ctsk). LncRNA‐Jak3 knockdown abolished the formation of mature osteoclasts induced by MSU.^137^

## 6 LNCRNAS AND KASHIN‐BECK DISEASE (KBD)

KBD is an endemic, teratogenic osteochondropathy. Pathological features include degeneration and necrosis of articular cartilage and growth plates.^138^, ^139^, ^140^ The aetiology of KBD is linked to environmental factors,^141^, ^142^ and hereditary factors are also thought to be involved.^143^, ^144^, ^145^ Recent transcriptional analysis of mRNAs, miRNAs and lncRNAs, combined with proteomic data from patients with KBD and Keshan disease, has revealed novel cellular pathways that may be related to selenium‐related regulation of transcription.^146^

A rat model of KBD was established by using T‐2 toxin. The selenium level of serum, IL‐1β and TNF‐α levels, and MIAT and phosphorylated p65 (p‐p65) expression levels were all increased in each intervention group. After isolating primary epiphyseal chondrocytes, the researchers found that selenium treatment reversed T‐2 toxin‐induced chondrocyte damage. In general, overexpression of MIAT or T‐2 toxin treatment can lead to inflammatory response, apoptosis and death. The activation of NF‐κB/p65 pathway and the increased expression of MIAT could be maintained by transfection of MIAT siRNA and selenium treatment.^147^

Wu et al^148^ identified up/down‐regulated lncRNAs and mRNAs in KBD chondrocytes through microarray analysis. Correlation analysis of 343 lncRNAs and 292 mRNAs revealed the formation of 509 co‐expression network (CNC network) of coding and non‐coding genes. It was predicted that there were 11 target genes with cis‐regulated lncRNAs. Differentially expressed mRNAs in KBD played an essential role in ECM related biological events. At the same time, 34 mRNAs and 55 co‐expressed lncRNAs constituted a network affecting ECM. In the network, LAMA 4 and FBLN1 were the core genes with the highest significance. These findings indicate that lncRNAs may be involved in ECM destruction in KBD.

## 7 LNCRNAS AND SPINAL DISEASES

### 7.1 LncRNAs and Ankylosing spondylitis (AS)

AS is a systemic chronic disease with progressive development, characterized by chronic inflammatory responses in the sacroiliac joints and spine, and it belongs to RA. Several studies have shown that lncRNAs could be used as an independent diagnostic biomarker for AS, such as lncRNA AK001085, LINC00311, TUG1 and NKILA.^149^, ^150^, ^151^, ^152^ LncRNA MEG3 is a potential regulator in AS. It has anti‐inflammatory effects, partly by targeting miR‐146a. Overexpression of miR‐146a reversed the inhibitory effect of abnormally expressed MEG3 on inflammatory factors.^153^ Another study revealed that MEG3 promotes SOST expression to restrain the progression of AS by sponging let‐7i.^154^ H19 is overexpressed in AS patients and mediates the inflammatory process by acting as a ceRNA on the miR22‐5P‐VDR‐IL‐17A/IL‐23 axis.^155^ By down‐regulating the expression of LOC645166 in T cells of AS patients, and by inhibiting the recruitment of IKK complex to the K63‐linked polyubiquitin chain and upregulating the activation of NF‐kB, AS patients showed higher sensitivity to the stimulation of pro‐inflammatory cytokines or TLR ligand.^156^

### 7.2 LncRNAs and cervical spondylotic myelopathy (CSM)

CSM is a neurodegenerative disease. The main aetiology is progressive compression and degeneration of the spinal cord.^157^ The expression profiles of lncRNAs and mRNAs in rat CSM model were analysed by microarray. 17 lncRNAs (13 up and 4 down) and 18 mRNAs (13 up and 5 down) were found to be differentially expressed. According to the analysis of these results, the biological processes involved in the upregulation of RNA in CSM included cellular response to interferon, inflammatory response and innate immune response. By associating the differentially expressed mRNAs with lncRNAs, the researchers revealed that the disease may be involved in apoptosis, TNF and nod‐like receptor signalling pathways.^158^

### 7.3 LncRNAs and Intervertebral disc degeneration (IDD)

Unlike articular cartilage, the intervertebral disc (IVD) is a well‐wrapped and vascularless tissue that has three components: the nucleus pulposus (NP), annulus fibrosus (AF) and cartilaginous end plate (CEP). Nucleus pulposus is located in the centre of each disc and is highly hydrated and gelatinous, surrounded by the lateral annulus fibrosus.^159^, ^160^ IVD is the largest avascular structure in the body and the nerve endings only reach the inner ring.^161^ Due to these structural characteristics, IVD is prone to degeneration.^162^ At present, the aetiology of IDD is determined by genetic and environmental factors. Heredity is a major risk factor for IDD, as it is estimated that over 70% of cases are caused by genetics.^163^, ^164^ IDD is known to be driven by programmed cell death,^165^ deficiency in anabolic factors, release of inflammatory cytokines^166^, ^167^ and degradation of intervertebral disc matrix.^168^

Recently, Wan et al and Chen et al examined the expression of lncRNAs in human degenerative and normal NP samples using lncRNA‐mRNA microarray. They found 116 lncRNAs (67 up and 49 down) are differentially expressed, with absolute fold changes greater than ten.^169^, ^170^ LncRNA TRPC7‐AS1 directly targeted miR‐4769‐5p while miR‐4769‐5p directly targeted Hepsin (HPN) 3'UTR. Overexpression of miR‐4769‐5p inhibited HPN expression, suppressed NPC senescence, promoted NPC viability and ECM synthesis.^171^ SNHG6 can upregulate the expression of Bax, Caspase‐3 and p21 and reduce the expression of Bcl‐2 by targeting miR‐101‐3p, finally inhibiting cell proliferation and inducing cell apoptosis.^172^ Through the miR‑93/MMP2 pathway, PART1 may regulate ECM degeneration, cell proliferation and apoptosis of NP cells.^173^ Studies have shown that MP1DT can activate the mitochondrial apoptosis pathway of NPCs by down‐regulating Bcl‐2 and upregulating caspase‐3. The combined use of lnc‐MT1DP and miR‐365 can damage mitochondrial membrane, reduce mitochondrial function and ROS clearance ability, increase cell apoptosis and lead to lumbar disc herniation (LDH).^174^ ANPODRT partially protects human NPC from oxidative stress and apoptosis by inducing KEAP1‐Nrf2 dissociation, leading to the accumulation of Nrf2 protein and nuclear translocation, as well as the expression of Nrf2 target proteins (including HO1 and NQO1) in human NPCs.^175^

**FIGURE 2:**
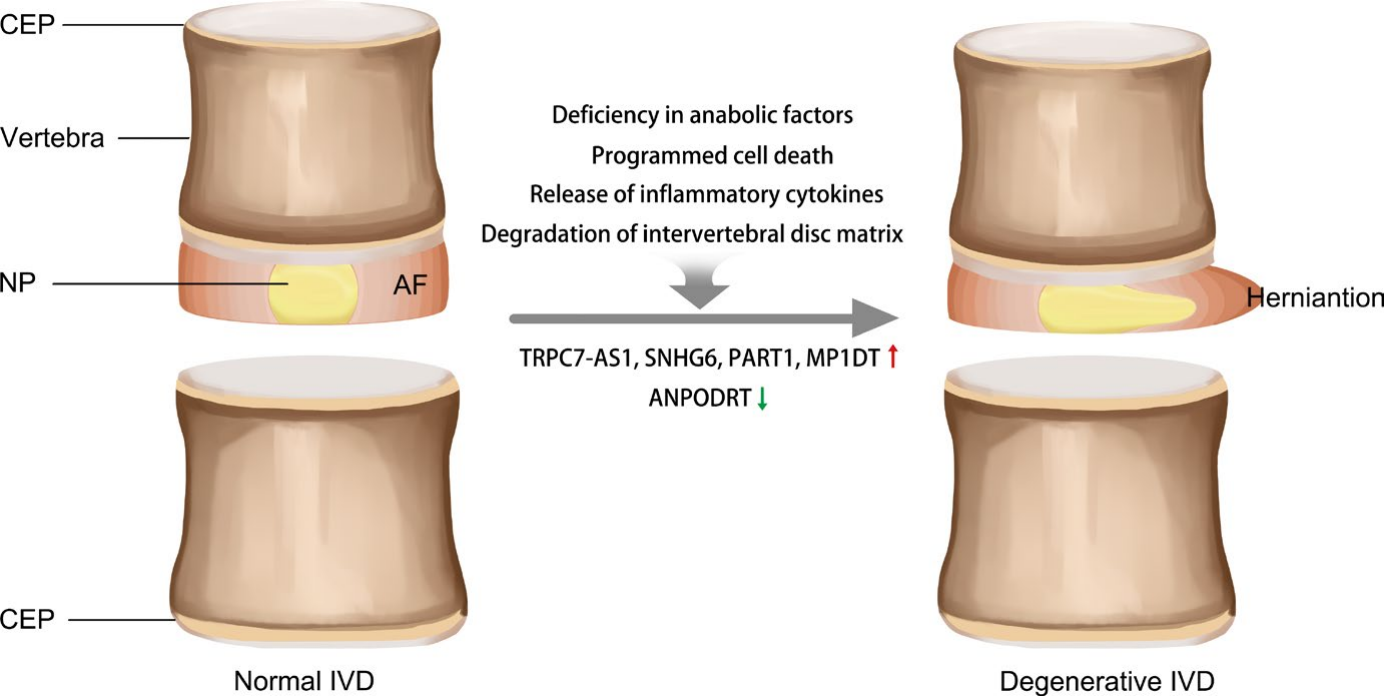
How does normal IVD become degenerative IVD. LncRNAs and four factors affecting the transition from normal to degenerated IVD. IVD consists of three distinct regions: NP, AF and CEPs. Red arrows indicate upregulation, and green arrows represent downregulation

Here, we focus on the roles and functions of the newly discovered lncRNAs in IDD (Figure 2). LncRNAs that appear in these published reviews^160^, ^176^ are not included in this section.

## 8 LNCRNAS AND MUSCLE DISEASES

Alterations in myogenesis and regeneration can lead to many muscle disorders (including muscle hypertrophy, muscular dystrophy and sarcopenia). Abnormal expression of lncRNAs is related to a variety of muscle diseases. Rescuing its normal expression in skeletal muscle can reduce the phenotype of the disease.

### 8.1 LncRNAs in human muscle disease

Among all types of muscular dystrophy, one of the most common and severe disorders is Duchenne muscular dystrophy (DMD). DMD, which involves multiple lncRNAs, is caused by a dysfunctional dystrophin protein. Some lncRNAs inhibit the expression of dystrophin mRNA subtypes by interacting with the dystrophin promoter.^177^, ^178^ In a recent study, LncRNA H19 was shown to bind with dystrophin. And H19 inhibited E3‐ligase‐dependent polyubiquitination and subsequent protein degradation at Lys 3584 (referred to as Ub‐DMD). DMD and BMD (Becker MD) are considered to be X‐linked recessive. Intra‐frame deletion of BMD and non‐silent mutation of DMD (C3340Y) lead to deficiency in the ability of the protein to interact with H19, resulting in elevated Ub‐DMD levels and degradation of dystrophic proteins. The discovery of H19 lncRNA as a dystrophin stabilizer may prove to be the missing link in the successful development of salvage therapies for DMD.^179^, ^180^ LncRNA44s2 could be involved in muscle differentiation process. Study in human primary myoblasts from BMDΔ45‐55 patients revealed a possible involvement of lncRNA sequences localized in intron 44 and 55 in myogenesis. Finally, it could be a potential biomarker for monitoring the development of DMD/BMD disease.^181^

Idiopathic inflammatory myopathy (IIM) includes myasthenia and myositis. In IBM and JO‐1 myositis patients, 16 lncRNAs including lncMyoD, MALAT1 and H19 were differentially expressed.^182^

### 8.2 lncRNAs in muscle hypertrophy and atrophy

LncRNAs in muscle atrophy and hypertrophy are also the focus of our attention. Studies have shown that the main causes of muscle hypertrophy are increased intracellular RNA and protein synthesis and decreased protein degradation. The equilibrium between protein synthesis and degradation is regulated by a number of regulators and pathways, including the mTOR, IGF and AMPK pathways, myostatin and myogenetic regulators.^11^ Skeletal muscle hypertrophy is positively regulated by the BMP7 signalling pathway through activation of Smad1/5.^183^ Moreover, muscle hypertrophy requires activation of satellite cells.^184^, ^185^ In myogenic differentiation, c‐Myc plays an important regulatory role. In addition to regulating protein‐coding genes, it also regulates the expression of non‐coding RNA to modulate myoblast differentiation via directly regulating the transcription of many MyomiRs. Luo et al suggested that linc‐2949 and linc‐1369 act as MyomiR sponges and regulate myoblast differentiation and proliferation.^186^ The evolutionarily conserved lncRNA linc‐MYH modulates the composition of the INO80 chromatin remodelling complex in muscle stem cells (MuSCs) and prevents interaction with WDR5 and transcription factor YY1. Linc‐MYH is expressed in proliferating myoblasts but not in resting MuSCs. So researchers infer that the degree of myoblastic proliferation has a decisive effect on the size of the quiescent MuSC.^187^

Muscular atrophy is the most common muscle disorder in humans and is accompanied by myophagism and muscle weakness.^188^ Li et al found that in various types of muscle atrophy models, lncRNA muscle atrophy‐related transcripts (lncMAAT) play different roles and regulate different genes through trans and cis regulation modules (trans:miR‐29b/SOX6 axis; cis:neighbouring gene Mbnl1).^189^ Lnc‐ORA inhibited skeletal muscle myogenesis via regulating acting miR‐532‐3p/PTEN/PI3K/AKT axis. In addition, LNC‐ORA interacted with IGF2BP2 (insulin‐like growth factor 2 mRNA‐binding protein 2) to influence the stability of myogenetic genes.^190^ SMUL regulates myogenesis and muscle atrophy via TGF‐β/Smad pathway. The mechanism is SMUL's inhibition of Smurf2 production through NMD (nonsense‐mediated mRNA decay).^191^ Finally, miR22HG induces the maturation of miR‐22‐3p, which inhibits its target HDAC4 (histone deacetylase 4), thereby increasing downstream MEF2C (myocyte enhancing factor 2C), and ultimately promoting myoblast differentiation.^192^

In conclusion, the current research focuses on muscle development after birth and growth, muscle hypertrophy and atrophy. LncRNAs related to muscle hypertrophy and atrophy are summarized in Table 4. We excluded the lncRNAs that appeared in these published reviews.^11^, ^193^, ^194^ In the near future, studies on muscle and lncRNAs will be oriented towards embryonic muscle generation and development, muscle fibre transformation, muscle function and movement, muscle ageing and metabolism, and muscle tumours.

**TABLE 4:** Summary of the roles of lncRNAs in muscle hypertrophy and atrophy

LncRNAs	Expression	Targets	Study models	Cellular process	Reference
Linc‐2949/linc‐1369	–	miR‐206 and miR‐1; c‐Myc	CPMs and chicken breast muscle tissue	Myoblast differentiation (2949, −) (1369, +)	^186^
Linc‐MYH	Up	INO80 chromatin remodeler complex/WDR5/YY1	Mice skeletal muscle tissue and MuSCs	Myoblast proliferation (+)	^187^
lncMAAT	Down	Trans: miR‐29b/SOX6; Cis: Mbnl1	Mice muscle tissue and C2C12 Mice myoblasts cell line	Muscle atrophy (−)	^189^
Lnc‐ORA	Up	miR‐532‐3p, PTEN/PI3K/AKT, IGFBP2	C2C12 cell line and aged mice muscle tissue	Myoblast proliferation (+) and differentiation (−)	^190^
SMUL	Up	SMURF2/NMD; TGF‐β/SMAD	CPMs and chicken muscle tissue	Myoblast proliferation (+), differentiation (−) and skeletal muscle atrophy (+)	^191^
miR22HG	Up	miR‐22‐3p/HDAC4	Mice skeletal muscle tissue; C2C12 and HEK293T cell lines	Myoblast differentiation and regeneration of skeletal muscle (+)	^192^

Abbreviations: CPMs, chicken primary myoblasts; IGFBP2, insulin‐like growth factor 2 mRNA‐binding protein 2; lncMAAT, lncRNA muscle‐atrophy‐associated transcript; Lnc‐ORA, obesity‐related lncRNA; Mbnl1, muscleblind‐like 1; MuSCs, Muscle stem cells; MYH, fast myosin heavy chain; SMUL, nonsense‐mediated mRNA decay; SOX6, SRY‐box 6; WDR5, WD‐40 repeat protein 5.

## 9 CONCLUSIONS AND FUTURE PERSPECTIVES

In this review, we summarized the functions and regulatory mechanisms of lncRNAs involved in the occurrence and progression of musculoskeletal disorders. LncRNAs have been found to participate in the regulation of chronic musculoskeletal disorders under various pathological conditions. Current studies mainly point to the interaction axis between lncRNA and miRNA and downstream molecules. More studies are urgently needed to investigate the underlying mechanism, such as the binding sites and ways of targeting downstream molecules, and whether there are multiple binding sites.

Furthermore, as described in this paper, some regenerative therapies involving stem cells are also associated with lncRNAs, such as mesenchymal stem cells (MSCs), which have been used in OA and IDD^195^ therapy to assist tissue regeneration and exosome secretion. This is also one of the research hotspots. And the role of lncRNAs in the regulation of intracellular or endochondral ossification and muscular dystrophy remains to be further studied. Finally, the interactions between circRNAs, lncRNAs, miRNAs and target genes also have considerable research potential.

Another area of active study is the post‐transcriptional modifications of lncRNAs. Post‐transcriptional modifications of RNA have been described in many sequencing‐based transcriptome studies. Three major modifications include pseudouridine (Ψ), N6‐methyladenosine (m6A) and 5‐methylcytosine (m5C).^196^, ^197^ Although the chemical modification of lncRNAs in other fields (eg, oncology) has suggested that its presence is important for the function of lncRNAs. But to date, no transcriptome changes have been reported to be associated with musculoskeletal diesease. Obviously, chemical modifications of RNAs are new areas of studying lncRNA functions.

Translational research on lncRNAs and musculoskeletal diseases will continue to flourish, in part due to our improving understanding of the functions of lncRNAs and the increasingly available practical methods to identify the functional domains of lncRNAs. Our understanding of the roles of lncRNAs in musculoskeletal disorders will lead to the development of new strategies to improve their clinical management.

## CONFLICT OF INTEREST

The authors declare that they have no competing interests.

## AUTHOR CONTRIBUTIONS

HH, DX and JL conceptualized the review; HH and DX searched the literature; HH wrote the draft of the manuscript; HH, QZ and HL prepared the figures and tables; all the authors critically reviewed and edited the manuscript. All authors read and approved the final manuscript.

## Data Availability

Research data are not shared.
